# Development of a nomogram to predict negative postoperative behavioral changes based on a prospective cohort

**DOI:** 10.1186/s12871-023-02228-4

**Published:** 2023-08-04

**Authors:** Lijing Li, Jianmin Zhang, Jiayi Li, Yi Ren, Zhengzheng Gao, Jia Gao, Fuzhou Zhang, Fang Wang, Tiehua Zheng

**Affiliations:** 1 Department of Anesthesiology, Beijing Children’s Hospital, Capital Medical University, National Center for Children’s Health, No. 56 South Lishi Road, Xicheng District, Beijing, 100045 China; 2 Department of Urology, Beijing Children’s Hospital, Capital Medical University, National Center for Children’s Health, Beijing, 100045 China

**Keywords:** Negative postoperative behavioral changes, Regional cerebral oxygen saturation, Prediction model, General anesthesia, Children

## Abstract

**Background:**

It is believed that negative postoperative behavioral changes (NPOBC) is associated with negative perioperative outcomes in children. The importance of development of a predictive model of NPOBC was noted. This study aims to identify potential risk factors develop a nomogram to predict NPOBC on postoperative day 3 based on a prospective cohort.

**Methods:**

A prospective observational study was conducted on children(American Society of Anesthesiologists I ~ III) aged 2 ~ 12 years who underwent selective surgery under general anesthesia between September 2022 and February 2023. The patient’s clinical data were analyzed. The method of measuring NPOBC is with the The Posthospital Behaviour Questionnaire (PHBQ), and all of children remained hospitalized at the time of assessment. The enrolled patients were categorized into the NPOBC group and the non-NPOBC group according to if children developed NPOBC on postoperative day 3. Univariate and multivariate logistic regression analyses were performed to identify independent risk factors and develop the nomogram to predict NPOBC. Internal validation was performed using the parametric bootstrapping method.

**Results:**

One hundred ninety-two patients were enrolled in the study, 44.8% (86/192 patients) of children developed NPOBC on postoperative day 3. Univariate and multivariate logistic regression analysis demonstrated that the Pediatric Anesthesia Behavior (PAB) score (OR: 1.23, 95%CI: 1.14–1.33), cerebral desaturation (OR: 1.16, 95%CI: 1.02–1.32), and postoperative pain score (OR: 1.07, 95%CI: 1.02–1.13) were independent predictors for NPOBC on postoperative day 3 (*P* < 0.05). They were used to develop the prediction model. The calibration curve demonstrated satisfied discrimination and calibration of the prediction model. The model presented with good discriminative ability (area under the receiver operating characteristic curve: 0.762 [95%CI: 0.691—0.833]). The decision curve analysis also revealed the great clinical utility of the nomogram.

**Conclusion:**

Based on our prospective observational study, pre-anesthesia patients with higher PAB scores, presence of cerebral desaturation, and higher postoperative pain score were more likely to develop NPOBC on postoperative day 3. We established and validated a nomogram for predicting NPOBC, which could help assess patients individually, identify high-risk groups of NPOBC and improve patient prognosis.

**Trial registration:**

Chinese Clinical Trial Registry, ChiCTR‐2,200,059,776. Registered 11 May 2022.

**Supplementary Information:**

The online version contains supplementary material available at 10.1186/s12871-023-02228-4.

## Introduction

Based on the principle of personalized medicine, protecting children’s perioperative mental health is gradually being emphasized [1]. The perioperative period is a stressful process for children, complex processes are involved in predicting critical events during the perioperative period and are frequently followed by negative postoperative behavioral changes (NPOBC) [2]. The Posthospital Behaviour Questionnaire (PHBQ) is currently used as the standard measure for detecting NPOBC [3, 4]. NPOBC include general anxiety, nightmares, eating disorders, temper tantrums, and enuresis, with the prevalence ranging from 29.8% to 43% on postoperative day 14 and from 16 to 32% on postoperative day 30 [5–8]. Although previous studies demonstrated that risk factors such as younger age, female sex, type of surgery, ethnic variations, preoperative anxiety, and intraoperative decrease in cerebral regional oxygen saturation (rScO_2_) had been associated with NPOBC, its etiology remains unclear [9–12]. It is believed that NPOBC is associated with negative prognosis, which could affect children’s emotional and cognitive development [2]. As not all children will need routine pharmacological or behavioral intervention, it is important to develop a predictive model of NPOBC [11].

In the present study, we prospectively collected patients’ data, analyzed the characteristics of patients who developed NPOBC on postoperative day 3, sought independent risk factors, and developed a predictive model. We expect the nomogram can help clinicians assess patients individually, identify high-risk patients of NPOBC, give early intervention to decrease adverse effects, and improve patient prognosis.

## Methods

### Participants

This prospective observational study was conducted at the National Center for Children’s Health, Beijing Children’s Hospital, China, over 6 months between September 2022 and February 2023. The study was registered at the Chinese clinical trial registry (ChiCTR‐2,200,059,776). The Ethics Committee of Beijing Children's Hospital, Capital Medical University has approved the use of fully anonymized cohort data for research (ID: 2021-E-114-Y). All children's guardians provided written informed consent.

Patients between the ages of 2 and 12 years, with American Society of Anesthesiologists (ASA) physical status I to III and scheduled for elective noncardiac surgery under general anesthesia for more than 60 min, with mechanical respiratory assistance, were included in the study. The exclusion criteria were: a. Children with previous neuropsychiatric disorders; b. Emergency surgery; c. Monitoring site with skin lesions or rash; d. Children who need to be transferred to Intensive Care Unit (ICU) for further treatment after surgery, e. Refusal by the parents to participate.

### Assessment

The primary outcome was the presence of NPOBC on postoperative day 3. NPOBC was assessed by the PHBQ (Supplemental Table 1), the five options, much less, less, same, more, and much more, were awarded scores of − 2, − 1, 0, 1, and 2, respectively. The total scores were obtained by summing the score of each item. we identified PHBQ scores ≥ 7 as substantial behavioral changes and defined to NPOBC [5]. Children’s behavior and mood state before the induction of anesthesia was evaluated by the Pediatric Anesthesia Behavior (PAB) score (Supplemental Table 2); the higher the score, the higher the level of anxiety [13]. The Pediatric Anesthesia Emergence Delirium (PAED) score was used to assess whether a child was experiencing delirium after general anesthesia (Supplemental Table 3); the onset of emergence delirium (ED) was defined as the first evaluation for each patient with a PAED score ≥ 10 [1, 14]. The face, legs, activity, crying, and consolability (FLACC) behavioral scale was used to quantify the pain behavior of children ≤ 7-year-old. (Supplemental Table 4) [15]. The visual analog scale (VAS) was used for children older than 8-year-old. Children were asked to score their pain on a slide rule of 10 cm in length,.and the extreme 0 indicates ‘no pain’, 10 cm indicates the worst pain imaginable (Supplemental Table 5) [16]. Both postoperative pain scale score greater than 4 pts indicates the presence of pain [1]. We followed up the postoperative pain twice on the first and second days after surgery, and recorded the scores separately. The average score of the two days was taken for analysis.

Parents completed the PHBQ on postoperative day 3 in order to avoid the influence of first two days agitation, significant pain or nausea on questionnaire response. In addition, all of children remained hospitalized at the time of assessment, and the doctor visits the ward for postoperative follow-up, which can explain to parents how to fill out the questionnaire and discover adverse behavior in hospitalized children.

### Anesthesia management

All patients will be hospitalized the day before surgery and undergo routine preoperative examinations. One day preoperatively, patients were identified through hospital surgical schedules. The routine preoperative checkup was performed, and written informed consent was obtained from the guardians of the patients. The standardized anesthesia protocol was provided by professional pediatric anesthesiologists. All children are fasted from solid food for six hours and from water two hours before surgery, and intravenous access was established in the ward.

The preoperative anxiety of children was measured upon entrance into the operation room and during induction by a trained pediatric anesthesiologist using the PAB Score. The medium sensor (FORE-SIGHT ELITE Cerebral Oxygen Saturation Monitor; NIRS, CAS Medical Systems Inc, Branford, CT) was attached to the clean and dry forehead above the eyebrows of the child before induction of anesthesia to get the baseline value of rScO_2_. The heart rate (HR), mean arterial pressure (MAP), rScO_2_, body temperature, and bispectral index (BIS) values of the children were continuously monitored. Anesthesia induction was performed with propofol 2 to 3 mg/kg, sufentanil 0.3 to 0.5 μg /kg, and cisatracurium 0.1 mg/kg to facilitate endotracheal intubation. After tracheal intubation, an anesthesia machine was connected to control the breathing. The tidal volume was 6 to 8 mL/kg, and the respiratory rate was 16 to 24 times/min. The above parameters were adjusted according to end-tidal carbon dioxide (PetCO_2_) at 35–45 mmHg during the operation, with an inhaled oxygen concentration of 50 to 70%. During the operation, the BIS value was controlled within 40 to 60 to ensure an appropriate depth of anesthesia. Propofol (6 to 10 mg/kg/h) and remifentanil (0.2 to 0.4 μg/kg/min) were used for anesthesia maintenance, with the dose adjusted to depth of anesthesia requirements (HR and systolic blood pressure [SBP] changed within 20% of baseline values). The body temperature was monitored using a nasopharynx thermometer and was kept between 36.0 and 37.5℃ using a warm air blanket. Surgical and anesthesia duration and adverse events were documented.

After surgery, the children were transferred to the post anesthesia care unit (PACU). The child was scored by the same trained observer using the PAED form and was escorted back to the ward for further observation after the child’s status stabilized.

### Data collection

Data were routinely collected using a standardized electronic anesthesia system (Docare, MedicalSystem Company). Information collected included demographic data including age, sex, weight, operation time, and operation type (scoliosis, fracture, abdominal tumor, hypospadias, hydronephrosis, and biliary tract). Preoperative data including the PAB score, pre-anesthesia HR, pre-anesthesia SBP, pre-anesthesia diastolic blood pressure (DBP), pre-anesthesia MAP, and pre-anesthesia rScO_2_. Intraoperative data including hemorrhage, intraoperative urine, intraoperative input, mean PetCO_2_, anesthesia duration and extubation duration, and cerebral saturation value. Postoperative data including the PAED score (the incidence of ED), the FLACC behavioral scale or VAS score (degree and incidence of postoperative pain). The rScO_2_ values of the right and left frontal monitors were recorded. The pooled value of rScO_2_ (mean value of the left and right sides) was used for analysis. Cerebral desaturation was defined as a decrease in rScO_2_ of 10% or more from the basal value for at least 3 min in children [17]. The PHBQ was completed by parents on postoperative day 3. Patients were divided into the NPOBC group and the non-NPOBC group according to if children developed NPOBC on postoperative day 3.

### Construction and validation of the nomogram

The primary outcome of the study is the presence of NPOBC on postoperative day 3. The risk factors for the presence of NPOBC on postoperative day 3 were identified using univariate logistic regression, and varibles with P < 0.05 were included in the multivariate logistic analysis. A total score was calculated by analyzing the scores corresponding to each predictor variable in the nomogram, and a probability of NPOBC was calculated. Different methods were used to evaluate and validate the poformance of the prediction model. The calibration curve were drawn to reveal the reliability of the prediction model [18]. Nomograms were internally validated using parametric bootstrapping (B = 2000). A receiver operating characteristic (ROC) curve analysis was used to evaluate discrimination. The decision curve analysis (DCA) was used to assess the clinical utility of the nomogram [19].

### Statistical analyses

The sample size was estimated using PASS software (version 15.0). We assumed three or four independent risk factors would be used to predict NPOBC, and each factor requires at least 10 to 15 cases of primary outcomes to ensure the reliability of the estimation. Based on our previous results, assuming the incidence of NPOBC is between 22 and 52%. We accepted an α error of 0.05 and a β error of 0.2 in a bilateral contrast, and the sample size of 136 (30/0.22) was targeted. Assuming a 15% of possible withdrawals and loss of follow‐up, the calculated sample size was 170.

Statistical analyses in the present study were performed using R software (version 4.0.3, http://www.r-project.org). The normality was assessed by Kolmogorov–Smirnov test. Continuous variables are expressed as the mean ± standard deviation or median [interquartile interval, IQR]. The t-test was used for normally distributed continuous variables to assess the differences between the two groups, and the Wilcoxon rank-sum test was used for continuous variables with non-normal distribution. For categorical variables, Fisher's exact test and χ2 test were used. The regression analysis results were presented as odd ratios (OR) with 95% confidence intervals (CI). All statistical results were reported as two-tailed *P*-values, and < 0.05 was considered statistically significant.

## Results

We assessed 200 children for eligibility and enrolled 192 children for our study. Reasons for exclusion were: Lost contact with parents during follow-up (*n* = 2), and parents could not understand the scale (*n* = 6). Finally, 192 children included the study. Table 1 summarizes the enrollment data and demographic characteristics of the 192 patients. The incidence of NPOBC on postoperative day 3 was 44.8% (86 cases). There were no significant statistical differences in age, gender, weight, operation time, operation type between the two groups. Patients in the NPOBC group have the higher PAB score than the non-NPOBC group (*P* < 0.001). For pre-anesthesia variables, there were no significant statistical differences in HR, SBP, DBP, MAP, and rScO_2_ (*P* > 0.05). As for intraoperative variables, there were no significant statistical differences in intraoperative urine, intraoperative input, mean PetCO_2_, anesthesia duration, and extubation duration (*P* > 0.05). However, the incidence of cerebral desaturation in the NPOBC group was 58.1% (50 cases), significantly higher than in the non-NPOBC group (38 cases, 35.8%, *P* = 0.003). Postoperatively, the higher PAED scores in the NPOBC group were observed than that in the non-NPOBC group, but there was no statistical significance (7.00 [3.00;10.0] vs. 6.00 [3.00;8.00], *P* = 0.105). The incidence of emergence delirium in the NPOBC group was higher than in the non-NPOBC group (39.5% vs. 23.6%, *P* = 0.026). The higher postoperative pain score was shown in the NPOBC group than that in the non-NPOBC group (3.50 [2.00;4.00] vs. 2.50 [1.50;3.00], *P* < 0.001), and the incidence of postoperative pain in the NPOBC group was higher than the non-NPOBC group (44.2% vs. 18.9%, *P* < 0.001).

**Table 1:** Characteristics of participants with or without NPOBC on postoperative day 3

Variable	Non-NPOBC group (*n* = 106)	NPOBC group (*n* = 86)	*P*-value
Age (years)	5.00 [3.00;8.00]	4.10 [3.00;7.00]	0.417
Gender, n (%):			0.051
male	48 (45.3%)	52 (60.5%)	
female	58 (54.7%)	34 (39.5%)	
Weight (kg)	19.0 [14.9;28.8]	18.0 [14.8;24.5]	0.132
Operation type, n (%):			0.917
scoliosis	34 (32.1%)	30 (34.9%)	
fracture	15 (14.2%)	14 (16.3%)	
abdominal tumor	20 (18.9%)	14 (16.3%)	
hypospadias	9 (8.49%)	8 (9.30%)	
hydronephrosis	19 (17.9%)	11 (12.8%)	
biliary tract	9 (8.49%)	9 (10.5%)	
PAB score, n (%):			** < 0.001**
1 pts	67 (63.2%)	24 (27.9%)	
2 pts	27 (25.5%)	26 (30.2%)	
3 pts	12 (11.3%)	36 (41.9%)	
Pre-anesthesia HR (mmHg)	103 (13.3)	105 (11.8)	0.313
Pre-anesthesia SBP (mmHg)	100 [96.0;110]	100 [95.0;107]	0.655
Pre-anesthesia DBP (mmHg)	60.0 [55.0;70.0]	61.0 [55.0;68.0]	0.438
Pre-anesthesia MAP (mmHg)	73.0 [70.0;80.0]	74.0 [69.2;80.8]	0.644
Pre-anesthesia rScO_2_ (mmHg)	84.0 [80.6;87.0]	84.2 [80.5;87.4]	0.611
Hemorrhage (ml)	10.0 [5.00;185]	17.5 [5.00;140]	0.372
Intraoperative urine (ml)	200 [57.5;385]	250 [60.0;450]	0.341
Intraoperative input (ml)	650 [300;1050]	750 [362;1100]	0.381
Mean PetCO_2_ (mmHg)	34.0 [32.0;36.0]	33.0 [32.0;35.0]	0.276
Anesthesia duration (min)	178 [125;250]	198 [126;269]	0.358
Extubation duration (min)	19.0 [15.0;23.0]	16.0 [13.0;21.0]	0.080
PAED score (pts)	6.00 [3.00;8.00]	7.00 [3.00;10.0]	0.105
Emergence delirium, n (%):			**0.026**
no	81 (76.4%)	52 (60.5%)	
yes	25 (23.6%)	34 (39.5%)	
Postoperative pain score (pts)	2.50 [1.50;3.00]	3.50 [2.00;4.00]	** < 0.001**
Postoperative pain, n (%):			** < 0.001**
no	86 (81.1%)	48 (55.8%)	
yes	20 (18.9%)	38 (44.2%)	
Cerebral desaturation, n (%):			**0.003**
no	68 (64.2%)	36 (41.9%)	
yes	38 (35.8%)	50 (58.1%)	

Bold values indicate statistical significance

*NPOBC* negative postoperative behavioral changes, *PAB* pediatric anesthesia behavior, *HR* heart rate, *SBP* systolic blood pressure, *DBP* diastolic blood pressure, *MAP* mean arterial pressure, *PetCO*_*2*_ endtidal carbon dioxide, *rScO*_*2*_ regional cerebral oxygen saturation, *PAED* pediatric anesthesia emergence delirium score

## Development and validation of the prediction model for NPOBC on postoperative day 3

**Fig. 1:**
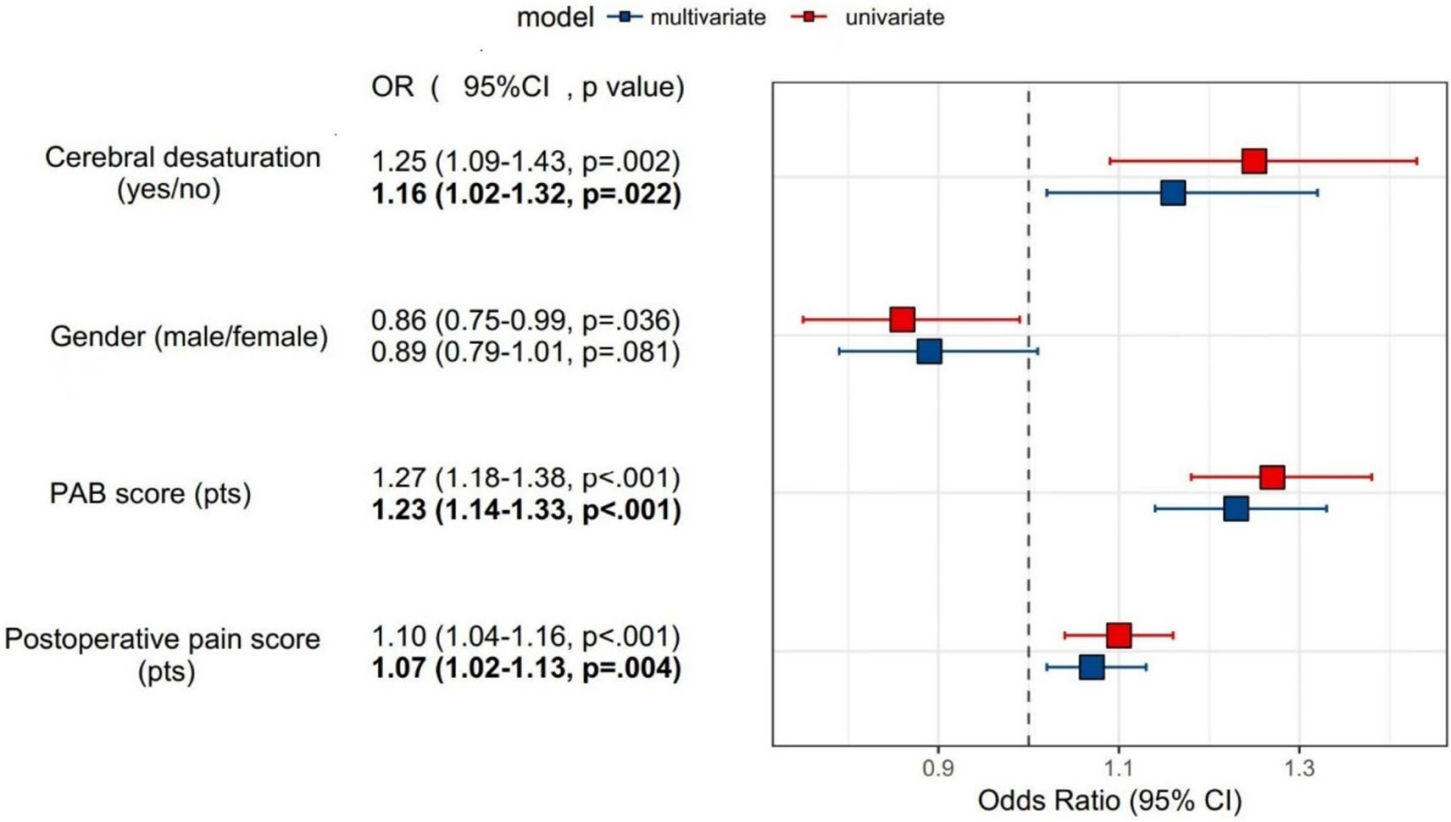
Logistic regression analysis on variables for the prediction of NPOBC on postoperative day 3. NPOBC, negative postoperative behavioral changes; PAB, pediatric anesthesia behavior; OR, odd ratios; CI confidence intervals

**Fig. 2:**
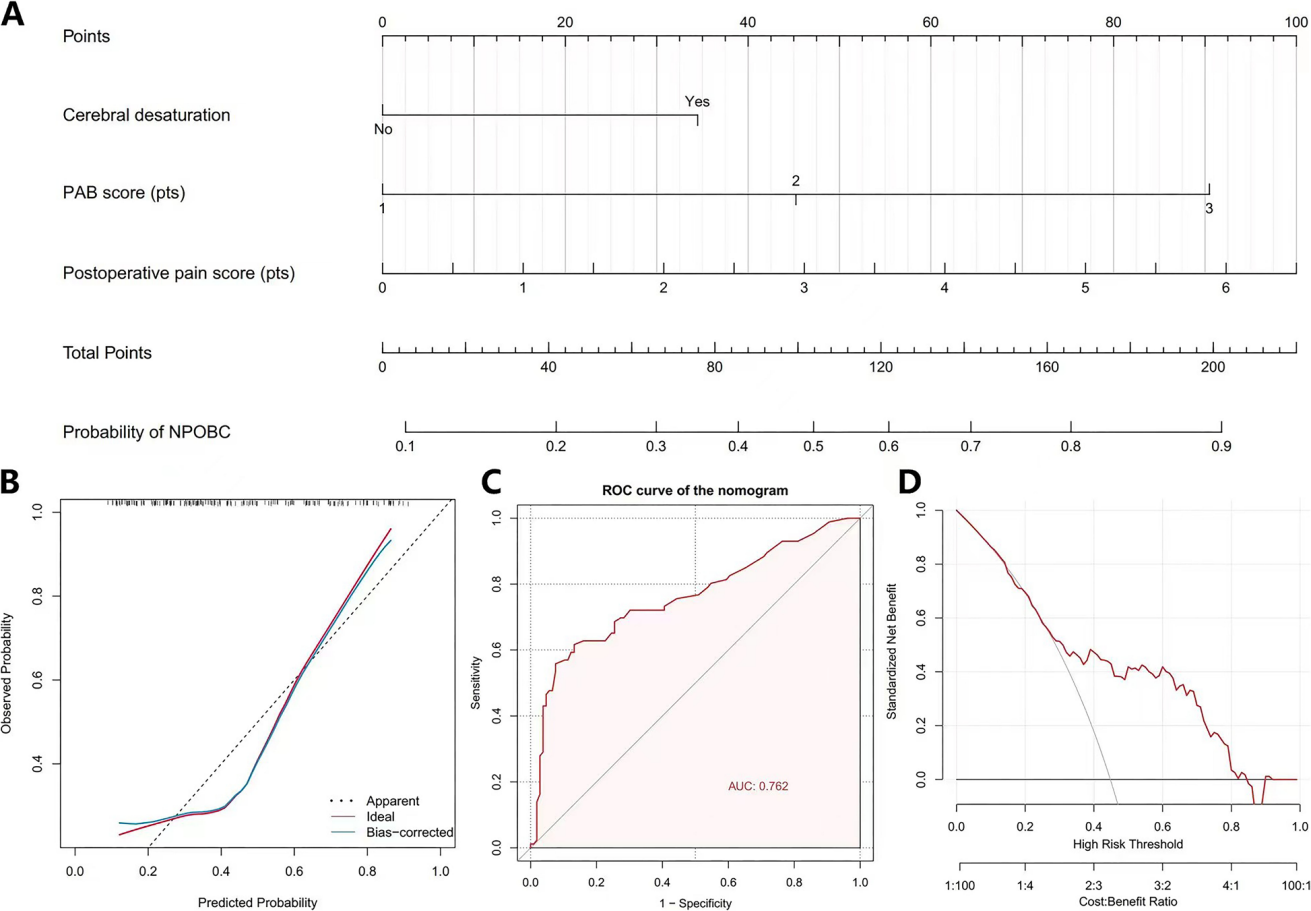
Development and validation of the nomogram predicting NPOBC on postoperative day 3. Development and validation of the nomogram predicting NPOBC on postoperative day 3. **A** Nomogram predicting NPOBC on postoperative day 3. The nomogram was constructed with three factors (Cerebral desaturation, PAB score, and postoperative pain score) identified by univariate and multivariate analyses. **B** Calibration curve of the prognostic nomogram model. The Y-axis scale represents the actual value probability of NPOBC on postoperative day 3 and the X-axis scale represents the predicted value calculated by the model. The dotted grey line represents an ideal model while the red line represents the nomogram’s prediction performance. **C** Receiver operating characteristic (ROC) curve of the prognostic nomogram model, the area under the curve (AUC) value reflected the discrimination performance of the model. **D** The decision curve analysis (DCA) curve of the model calculating the net benefit at different threshold probabilities. NPOBC, negative postoperative behavioral changes; PAB, pediatric anesthesia behavior

The characteristics of patients were used to fit the univariate logistic regression model, and the variables with *P* < 0.05 were identified: gender (male/female), PAB score (1pts/2pts/3pts), cerebral desaturation (N/Y), postoperative pain score (pts) (Table 2). The multivariate logistic regression model included these four variables. As shown in Table 2 and Fig. 1, PAB score (OR: 1.23, 95%CI: 1.14–1.33), cerebral desaturation (OR: 1.16, 95%CI: 1.02–1.32), postoperative pain score (OR: 1.07, 95%CI: 1.02–1.13) were independent predictors (*P* < 0.05) for NPOBC on postoperative day 3. Furthermore, these three predictors were used for developing the prediction model and for constructing the nomogram (Fig. 2A).

**Table 2:** Logistic regression analysis on variables for the prediction of NPOBC on postoperative day 3

Variable	Univariate analysis		Multivariate analysis	
	OR (95%CI)	*P*-value	OR (95%CI)	*P*-value
Age (years)	0.99 (0.97–1.01)	0.416		
Gender (male/female)	0.86 (0.75–0.99)	**0.036**	0.89 (0.79–1.01)	0.081
Weight (kg)	0.99 (0.99–1.00)	0.066		
Operation type: scoliosis	Ref	Ref		
fracture	1.01 (0.81–1.27)	0.901		
abdominal tumor	0.94 (0.77–1.17)	0.595		
hypospadias	1.00 (0.75–1.33)	0.989		
hydronephrosis	0.93 (0.74–1.16)	0.508		
biliary tract	1.03 (0.79–1.34)	0.817		
PAB score (1pts/2pts/3pts)	1.27 (1.18–1.38)	** < 0.001**	1.23 (1.14–1.33)	** < 0.001**^a^
Pre-anesthesia rScO_2_ (mmHg)	1.00 (0.99–1.02)	0.643		
Pre-anesthesia HR (mmHg)	1.00 (1.00–1.01)	0.399		
Pre-anesthesia SBP (mmHg)	1.00 (0.99–1.01)	0.581		
Pre-anesthesia DBP (mmHg)	1.00 (0.99–1.01)	0.627		
Pre-anesthesia MAP (mmHg)	1.00 (0.99–1.01)	0.929		
Hemorrhage (ml)	1.00 (1.00–1.00)	0.399		
Intraoperative urine (ml)	1.00 (1.00–1.00)	0.642		
Intraoperative input (ml)	1.00 (1.00–1.00)	0.526		
Mean PetCO_2_ (mmHg)	0.98 (0.96–1.01)	0.197		
Anesthesia duration (min)	1.00 (1.00–1.00)	0.156		
Extubation duration (min)	0.99 (0.98–1.00)	0.162		
Cerebral desaturation (N/Y)	1.25 (1.09–1.43)	**0.002**	1.16 (1.02–1.32)	**0.022**^a^
PAED score (pts)	1.02 (1.00–1.04)	0.080		
Postoperative pain score (pts)	1.10 (1.04–1.16)	** < 0.001**	1.07(1.02–1.13)	**0.004**^a^

Bold values indicate statistical significance. ^a^Factors included in the nomogram

*NPOBC* negative postoperative behavioral changes, *PAB* pediatric anesthesia behavior, *HR* heart rate, *SBP* systolic blood pressure, *DBP* diastolic blood pressure, *MAP* mean arterial pressure, *PetCO*_*2*_ endtidal carbon dioxide, *rScO*_*2*_ regional cerebral oxygen saturation, *PAED* pediatric anesthesia emergence delirium score, *OR* odd ratios, *CI* confidence intervals

The prediction model was validated by the 2000 repetitions of bootstrap sample corrections. According to the calibration curve of the nomogram, the predicted and observed probabilities of NPOBC are highly consistent (Fig. 2B). The AUC of the nomogram was 0.762 (95%CI: 0.691—0.833) for the prediction of NPOBC (Fig. 2C), indicating great discrimination of the prediction model. Furthermore, the DCA curve of the nomogram illustrated the strong clinical utility of the predictive model (Fig. 2D).

## Discussion

Children in critical stages of development are particularly vulnerable. Surgical stress response, intraoperative blood transfusion, a decrease in rScO_2,_ and other factors may cause negative changes in the perioperative period [20]. According to previous research, the incidence of NPOBC in the early postoperative period has been reported to vary from 24 to 80% [21]. The heterogeneity of the NPOBC incidence is perhaps due to the limited sample size, different diagnostic methods of NPOBC, or variations in the time that the behavior was assessed [7, 22]. A prospective study involving 198 children indicated that 38.8% children developed NPOBC on postoperative day 7 [9]. In Kain et al.'s study, 67%, 45%, and 23% of patients showed NPOBC on day 1, day 2, and two weeks after surgery [22]. The current study was conducted on a more general pediatric population, including children with ASA I and II undergoing non outpatient operations such as scoliosis, fractures, abdominal tumors, etc. In accordance with previous research, 44.8% of the children diagnosed with NPOBC during the third postoperative day used the 27-item validated PHBQ to diagnose the disease. NPOBC is detrimental to the treatment and recovery of the disease and can affect the emotional and cognitive development of the child, causing depression and anxiety in severe cases and threatening life and health [23]. Since the neurological system of pediatric patients is not well developed, they are more vulnerable to external influences. Therefore, it is important to establish an accurate and effective prediction model in pediatric patients to predict the occurrence of NPOBC individually.

NPOBC is associated with multiple risk factors, including young age, type of surgery, preoperative and induction anxiety in the preoperative periods, and method of inhalation anesthetic agent used [12, 24, 25]. In the current study, patients who developed NPOBC on postoperative day 3 have a higher PAB score, higher incidence of cerebral desaturation, higher incidence of emergence delirium, and higher incidence of postoperative pain than those in the non-NPOBC group (*P* < 0.05). After univariate and multivariate logistic regression analyses, the PAB score (OR: 1.23, 95%CI: 1.14–1.33), cerebral desaturation (OR: 1.16, 95%CI: 1.02–1.32), and The postoperative pain score (OR: 1.07, 95%CI: 1.02–1.13) were independent predictors for NPOBC on postoperative day 3 (*P* < 0.05), which are consistent with the previous studies [7, 26, 27].

NPOBC has previously been associated with preoperative anxiety in children [28, 29]. A prospective observational study was conducted by Beringer et al. on 102 children undergoing elective dental extractions under general anesthesia, demonstrating that distress during anesthesia induction was associated with NPOBC on postoperative days 1 and 7 [28]. In the present study, pre-anesthesia behavior and mood of the children were evaluated by the PAB score. Patients in the NPOBC group have a higher PAB score than the non-NPOBC group (*P* < 0.001). After univariate and multivariate logistic regression analyses, the PAB score was the independent risk factor for NPOBC on postoperative day 3(OR: 1.23, 95%CI: 1.14–1.33). Besides, patients in the NPOBC group have a higher postoperative pain score than that in the non-NPOBC group (3.50 [2.00;4.00] vs. 2.50 [1.50;3.00], *P* < 0.001), and higher incidence of postoperative pain was observed than those in the non-NPOBC group (P < 0.05), accordingly. Univariate and multivariate logistic regression analyses demonstrated that the The postoperative pain score (OR: 1.07, 95%CI: 1.02–1.13) was independent predictor for NPOBC on postoperative day 3 (*P* < 0.05). Our results are consistent with the findings of Luo et al. [10], Kain et al. [7], and Stargatt et al. [27], but inconsistent with the research of Kotiniemi et al. [30] and Power et al. [5]. According to Luo et al., the inconsistent results may be caused by the different pain assessment measures and different assessment times. For example, by using the FLACC scale, researchers may overestimate the pain severity due to the difficulty in distinguishing between pain [10].

Another independent risk factor for NPOBC on postoperative day 3 is the presence of cerebral desaturation (OR: 1.16, 95%CI: 1.02–1.32). There is no standardized, general absolute to define pathological brain region desaturation in children, perhaps partially attributable to the wide baseline variation of rScO_2_ in children, the presence of comorbidities, and the children’s immature, vulnerable brain was more vulnerable to the effects of the anesthetic [31, 32]. It is commonly acknowledged that intraoperative decreases in basal rScO_2_ values of 20% or more are harmful and are associated with postoperative cognitive dysfunction in adults [23]. However, children's regional cerebral oxygen saturation may reflect the balance between the consumption and the supply of oxygen of not only a local area under the forehead but of most of the brain due to a more immature self-regulating cerebral system [33, 34]. Previous studies demonstrated that even a decrease in rScO_2_ of less than 20% from baseline values might reflect high intraoperative bleeding or postoperative behavioral changes in children [35]. We speculate the reason is that the brain of children is immaturity and in the development stage and has different tolerance and regulation of hypoxia than adults. Thus, we referred to the standard of previous studies on children and defined rScO_2_ desaturation as more than 10% below baseline values for at least 3 min in the present study [17]. The rScO_2_ is believed to be affected by cerebral perfusion index, systemic oxygenation, and cerebral metabolism, which are possibly influenced by anesthesia [32, 36]. In the present study, patients were less likely to suffer from impaired cerebral oxygen extraction because their underlying physical condition was generally good, while they maintained sufficient oxygen saturation (SpO_2_ > 98%) and adequate body temperature and hemodynamics during general anesthesia.

Our nomogram included all three independent risk factors described above. To the best of our knowledge, this is the first study to build the nomogram with preoperative factors based on a prospective cohort of 192 patients and quantify the probability of NPONC individually. Parametric bootstrapping internally validated the established nomogram, which demonstrated good generalization and predictive performance. According to the calibration plot, the predicted and observed consequences were in agreement. ROC analysis revealed excellent discrimination between the nomogram and the NPOBC on postoperative day 3 (AUC = 0.762, 95%CI: 0.691—0.833). As the incidence of NPOBC in the early postoperative period reported in previous studies ranged from 24 to 80% [21]. In the present study, the incidence of NPOBC on postoperative day 3 was 44.8% (86 cases), DCA curves lay above none and all lines, demonstrating that the predictive model was clinically useful. We believe the nomogram is the first step toward an objective, individualized prediction of NPOBC in the early postoperative period. By identifying high-risk children of NPOBC, clinicians can improve prognosis of children.

The prospective design of this study may exclude some confounding factors. However, it is important to note that our study has some limitations. First, PHBQ is designed for assessing children’s posthospitalization and postoperative new onset behavioral changes and has a good profile of reliability and validity [37]. PHBQ used as the standard measure for detecting NPOBC, but it is typically used to evaluate children who are post-hospitalization, or have been discharged from the hospital. In this study, the children are on the hospital ward during assessment, which may lead to a high rate of NPOBC. NPOBC can prolong hospital stay [37], and we also hope to establish a predictive model for NPOBC during hospitalization in children to reduce incidence and shorten hospital stay. However, we have not found a more suitable evaluation method. Second, we did not attempt to control anesthetic regimen in this observational study, and this included a single center regional database, and the selection bias and the management of the perioperative period may likely be associated with NPOBC. Third, in the present study, we conducted on a more general pediatric population, including scoliosis, fracture, abdominal tumor, hypospadias, hydronephrosis, and biliary tract. Some of these procedures may have produced more pain than others. As postoperative pain has been demonstrated as the predictor of NPOBC, we will focus on the specific surgery type in our future study. Last, patients were unable to use self-assessment methods for postoperative pain assessment under 7 years old, we may have overestimated the pain severity due to the difficulty in distinguishing between discomfort and pain, by using the FLACC scale.

## Conclusion

In our prospective observational study, 44.8% of children (86/192 patients) developed NPOBC on postoperative day 3. Patients with higher PAB scores, presence of cerebral desaturation, and higher postoperative pain score were more likely to develop NPOBC on postoperative day 3. We innovatively established and validated a nomogram for predicting NPOBC. By using the nomogram, clinicians can make customized assessments of patients, identify patients at higher risk of NPOBC early on, and provide them with the care and support they need, thereby minimizing the likelihood of negative outcomes after surgery.

### Supplementary Information

**Additional file 1.**

## Data Availability

The key data are contained in the figures, tables, and additional files. The datasets used and/or analyzed during this study can be further obtained from the corresponding author, Jianmin Zhang, on reasonable request.

## Abbreviations

NPOBC: Negative postoperative behavioral change
PHBQ: Posthospital Behaviour Questionnaire
rScO_2_: Cerebral regional oxygen saturation
ASA: American Society of Anesthesiologists
ICU: Intensive Care Unit
PAB: Pediatric Anesthesia Behavior
PAED: Pediatric Anesthesia Emergence Delirium
ED: Emergence delirium
FLACC: Face, legs, activity, crying, and consolability
VAS: Visual analog scale
HR: Heart rate
MAP: Mean arterial pressure
BIS: Bispectral index
PetCO_2_: End-tidal carbon dioxide pressure
PACU: Post anesthesia care unit
SBP: Systolic blood pressure
DBP: Diastolic blood pressure
ROC: Receiver operating characteristic
DCA: Decision curve analysis
IQR: Interquartile interval
OR: Odd ratios
CI: Confidence intervals
