# Supplementary material for: Development of a nomogram to predict negative postoperative behavioral changes based on a prospective cohort
Source: BMC Anesthesiol. 2023 Aug 4;23:261. doi: 10.1186/s12871-023-02228-4 (PMC10401797; doi:10.1186/s12871-023-02228-4)

Supplemental Materials

Development of a Nomogram to Predict Negative Postoperative Behavioral Changes Based on A Prospective Cohort

Lijing Li MD, Jiayi Li MD, Yi Ren MD, Zhengzheng Gao MD， Jia Gao MD , Fuzhou Zhang MD, Fang Wang MD, Tiehua Zheng MD, Jianmin Zhang MD*

***Corresponding Author:** Jianmin Zhang, MD

Department of Anesthesiology, Beijing Children’s Hospital

No. 56 South Lishi Road, Xicheng District, Beijing 100045, China

Tel: +86 10 59616453

Fax: +86 10 59718710

E-mail: zhangjianmin@bch.com.cn

**Supplemental Table 1 Post-Hospitalization Behavioral Questionnaire**					
Score	-2	-1	0	1	2
	much less than before	less than before	same as before	more than before	much more than before
1. Does your child make a fuss about going to bed at night?					
2. Does your child make a fuss about eating?					
3. Does your child spend time just sitting or lying and doing nothing?					
4. Does your child need a pacifier?					
5. Does your child seem to be afraid of leaving the house with you?					
6. Is your child uninterested in what goes on around him (or her)?					
7. Does your child wet the bed at night?					
8. Does your child bite his (or her) finger nails?					
9. Does your child get upset when you leave him (or her) alone for a few minutes?					
10. Does your child need a lot of help doing things?					
11. Is it difficult to get your child interested in doing things (like playing games, with toys, and so on?)					
12. Does your child seem to avoid or be afraid of new things?					
13. Does your child have difficulty making up his (or her) mind?					
14. Does your child have temper tantrums?					
15. Is it difficult to get your child to talk to you?					
16. Does your child suck his (or her) fingers or thumbs?					
17. Does your child seem to get upset when someone mentions doctors or hospitals?					
18. Does your child follow you everwhere around the house?					
19. Does your child spend time trying to get or hold your attention?					
20. Is your child afraid of the dark?					
21. Does your child have bad dreams at night or wake up and cry?					
22. Is your child irregular in his (or her) bowel movements?					
23. Does your child have trouble getting to sleep at night?					
24. Does your child seem to be shy or afraid around strangers?					
25. Does your child have a poor appetite?					
26. Does your child tend to disobey you?					
27. Does your child break toys or other objects?					

Supplemental Table 2. The Pediatric Anesthesia Behavior Score

Score	Description of behaviour	
1	Happy	Calm and controlled. Compliant with induction
2	Sad	Tearful and/or withdrawn but compliant with induction
3	Mad	Loud vocal resistance (screaming or shouting) AND/OR Physical resistance to induction requiring physical restraint by staff and/or parents

Supplemental Table 3. The Pediatric Anesthesia Emergence Delirium Scale

1. The child makes eye contact with the caregiver.
2. The child's actions are purposeful.
3. The child is aware of his/her surroundings.
4. The child is restless.
5. The child is inconsolable.

Items 1, 2, and 3 are reversed scored as follows: 4=not at all, 3=just a little, 2=quite a bit, 1=very much, 0=extremely. Items 4 and 5 are scored as follows: 0=not at all, 1=just a little, 2=quite a bit, 3=very much, 4=extremely. The scores of each item were summed to obtain a total Pediatric Anesthesia Emergence Delirium (PAED) scale score. The degree of emergence delirium increased directly with the total score.

Supplemental Table 4. The face, legs, activity, crying, and consolability behavioral scale

Categories/Score	0	1	2
Face	No particular expression or smile	Occasional grimace or frown, withdrawn, disinterested	Frequent to constant frown, clenched jaw, quivering chin
Leg	Normal position or relaxed	Uneasy, restless, tense	Kicking, or legs drawn up
Activity	Lying quietly, normal position, moves easily	Squirming, shifting back and forth, tense	Arched, rigid, or jerking
Cry	No cry (awake or asleep)	Moans or whimpers, occasional complaint	Crying steadily, screams or sobs, frequent complaints
Consolability	Content, relaxed	Reassured by occasional touching, hugging, or being talked to, distractable	Difficult to console or comfort

Each of the five categories (F) Face, (L) Legs, (A) Activity, (C) Cry, (C) Consolability is scored from 0 to 2, which results in a total score between 0 and 10

Supplemental Table 5. The Visual Analog Scale

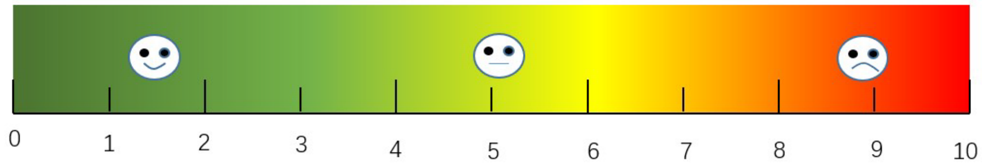

Supplement: Supplementary file 1 — Additional file 1. [file 12871_2023_2228_MOESM1_ESM.pdf]
